# Supplementary figures and images for: Simulation enabled search for explanatory mechanisms of the fracture healing process
Source: PLoS Comput Biol. 2018 Feb 2;14(2):e1005980. doi: 10.1371/journal.pcbi.1005980 (PMC5812655; doi:10.1371/journal.pcbi.1005980)

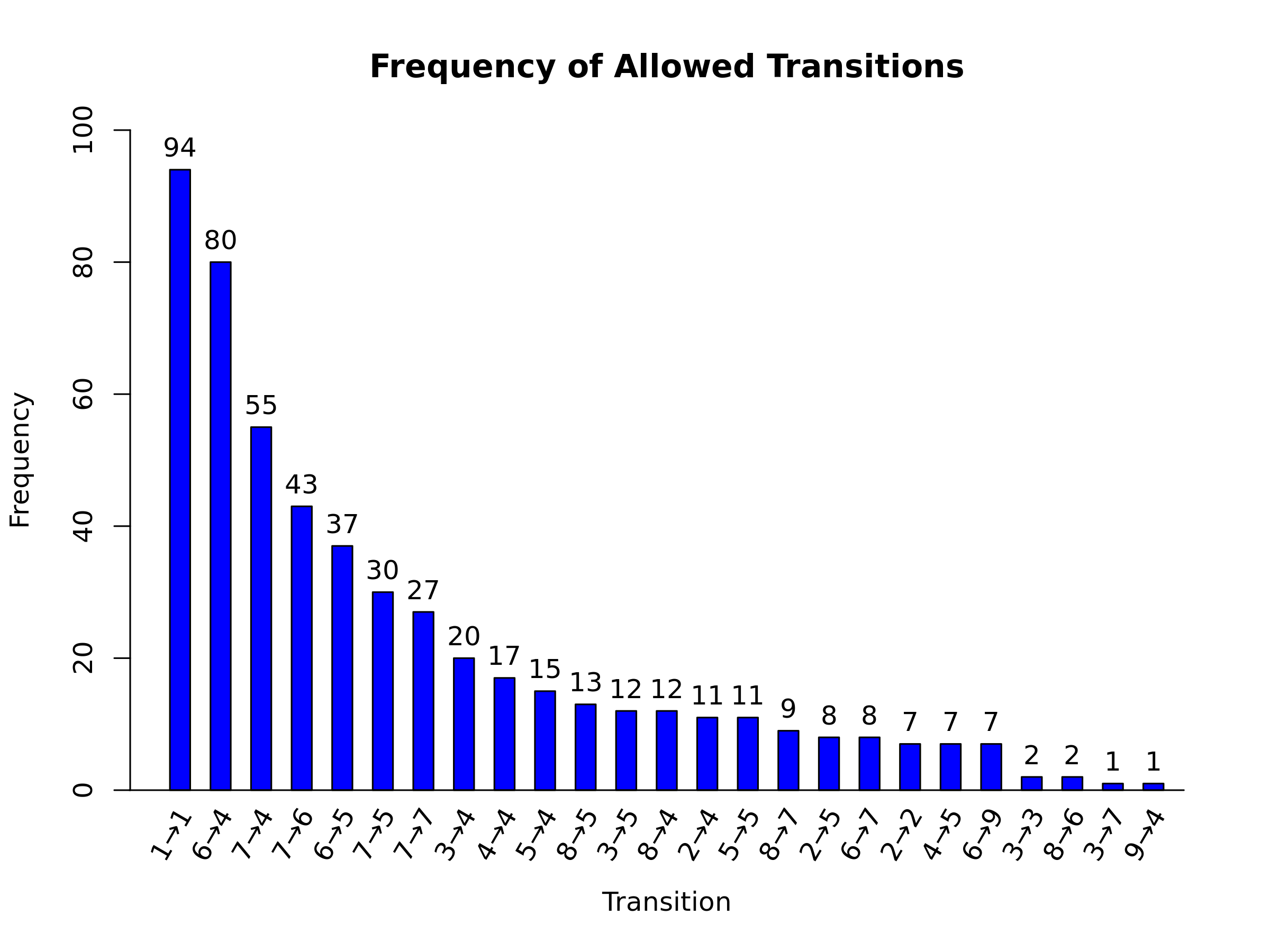

Supplement: S1 Fig — This plot depicts the frequency of the 25 allowed transition types, which include transitions where the final state was the same as the initial state. (TIF) [file pcbi.1005980.s001.tif]
