# Supplementary material for: Simulation enabled search for explanatory mechanisms of the fracture healing process
Source: PLoS Comput Biol. 2018 Feb 2;14(2):e1005980. doi: 10.1371/journal.pcbi.1005980 (PMC5812655; doi:10.1371/journal.pcbi.1005980)
Supplement: S1 Text — Forward/backward chaining reasoning strategies are described. Such strategies aid discovery of explanatory model mechanisms. Moving forward, we will rely on those strategies to expand Callus Analog to earlier (e.g., day-4) and later (e.g., day-14) stages of fracture healing. Mechanism is defined. To meet the definition of mechanism, the software mechanisms must exhibit the features listed in S1 Table within S1 Text. The table describes features that should be exhibited by an explanatory mechanism, along with the Callus Analog counterparts. (PDF) [file pcbi.1005980.s002.pdf]

S1 Text

Simulation Enabled Search for Explanatory Mechanisms of the Fracture Healing Process

Ryan C. Kennedy, Meir Marmor, Ralph Marcucio, and C. Anthony Hunt

Beyond demonstrating feasibility, limiting attention to just one target region in stage 3 of Fig 1 is an essential first step within our long-term strategy for discovering explanatory mechanisms. Doing so adheres to the important guideline in step 3 of the Iterative Refinement Protocol: take small steps.

A model mechanism as a plausible explanation of a phenomenon – A requisite for discussing mechanism-oriented biomedical models is adopting a definition for "mechanism." Over the past two decades, mechanism has emerged as a framework for thinking about fundamental issues in biology [1, 2]. Foremost, a mechanism is a real thing; it is concrete. Our working definitions are as follows. 1) A mechanism is a structure, system (e.g., biological, mechanical, software, chemical, electrical, and so on), or process performing a function in virtue of its component parts, component operations, and their organization (adapted from [3]), where the function is responsible for the phenomenon to be explained. 2) A mechanism comprises entities and activities organized in such a way that they are responsible for the phenomenon to be explained (adapted from [4,5]).

Braillard and Malaterre stated recently that a biological mechanism "...can be thought of as being composed of parts that interact causally … and that are organized in a specific way. This organization determines largely the behavior of the mechanism and hence the phenomena that it produces." [6] Accepting that point and given the first definition, it follows that a mechanical, software, chemical, electrical, or some other type of concrete mechanism can stand as a model mechanism that can prove useful in explaining biomedical phenomenon for which the actual mechanism remains to be elucidated. Craver posits that mechanistic biological models are intended to be explanatory, but he notes that, "some models sketch explanations but leave crucial details unspecified or hidden behind filler terms. Some models are used to conjecture a how-possibly explanation without regard to whether it is a how-actually explanation."[7]

It is also helpful to also have a description for the term "mechanistic" as used in the phrase "mechanistic model." Kaplan and Craver state that, "the line that demarcates [mechanistic] explanations from merely empirically adequate models seems to correspond to whether the model describes the relevant causal structures that produce, underlie, or maintain the explanandum phenomenon. This demarcation line is especially significant as it also corresponds to whether the model in question reveals (however dimly) knobs and levers that potentially afford control over whether and precisely how the phenomenon manifests" [8].

The increasing variety and sophistication of published mechanism-oriented and mechanism-based explanatory models reflect that biological mechanisms exhibit features that are not covered by the above definitions. Darden discusses how features of mechanisms often become necessary parts of adequate descriptions and representations of a mechanism [9]. She identifies five features of biological mechanism, listed in S1 Table, that often characterize mechanisms that adequately explain biological phenomena. The phenomenon to be explained is the essential first feature because the search for a mechanism-based model of explanation requires that the phenomenon be

clearly identified. Also, in biology, it is often the case that phenomena at a finer biological scale constitute the explanatory mechanism of the phenomenon of interest observed at coarser biological scale. Stated differently, the underlying finer details are often the entities and activities responsible for observable coarser behavior.

As alluded to in the Introduction, Darden describes a biological mechanism as a concrete and real system of entities and activities orchestrated so that it produces the phenomenon of interest. Darden argues that a biologically explanatory model mechanism will exhibit the features listed in S1 Table. Callus Analog Mechanisms exhibit those same features and examples, and are listed in S1 Table.

S1 Table | Five features of a biological mechanism (adapted from [9]): a biological mechanism exhibits all five. A computational mechanism-based model, such as Callus Analog, may strive to do the same.

**Mechanism Features Identified by Darden** [10]	**Callus Analog Examples**
**Phenomenon**	Fracture healing, more specifically the process of transforming Target Region initial state into a simulated Target Region final state
**Components**	
Entities and activities	Tissue-units; transition to another Tissue-object type
Modules	Tissue-units
**Spatial arrangement of components**	
Localization	Target Region, east of Bone
Structure	2D grid
Orientation	Direction to transition; probability gradients
Connectivity	Region of teal-blue interface
Compartmentalization	Gradients; vectors; Moore neighborhood
**Temporal aspects of components**	
Order	Allowed transitions; progressive changes of state
Rate	Transition rate (probability/time step)
Duration	Initial state to simulated final state
Frequency	Time steps
**Contextual locations**	Referent Region within callus
Location within a hierarchy	Callus, Referent Region, blue Tissue-units
Location within a series	Transitions that follows expansion

Mechanism discovery via forward/backward chaining – Darden explains that mechanism "discovery proceeds in stages of construction, evaluation, and revision. Each of these stages is constrained by what

is known or conjectured about what is being discovered. … Guidance in discovering mechanisms may be provided by the reasoning strategies of schema instantiation, modular subassembly, and forward/backward chaining" [10]. Insight into fracture healing mechanisms is still too sparse to support explanatory schema. However, we used ideas from modular subassembly and forward/backward chaining reasoning strategies to frame our starting approach and guide target region selection. Modular subassembly "involves reasoning about groups of mechanism components. One hypothesizes that a mechanism consists of known modules or types of modules. One cobbles together different modules to construct a hypothesized mechanism … Forward chaining uses the early stages of a mechanism to reason about the types of entities and activities that are likely to be found downstream. Backward chaining reasons from the entities and activities in later stages in a mechanism to find entities and activities appearing earlier" [10].

Longer term, we will need to employ variations of the forward/backward chaining strategy, and planning for that requires selection of a new target region. Once we attain a simulated healing process that can achieve our stage 6 objective for the day-10i tissue section, we envision subsequent iterative refinement work that extends simulated healing *forward* to achieve a *new* stage 6 objective for a day-14i tissue sections, for example, and subsequently *backward*, to achieve an another *new* stage 6 objective for a day-4i tissue sections, for example, as illustrated in Fig 4. Because the immediate objective of the healing process is different at different locations along a common healing path, we can be reasonably confident that requiring iterative refinement and improvement of Callus Analog mechanisms will enable achieving those two new objectives while continuing to achieve the stage 6 objective for the day-10i tissue section. There are additional considerations. The histological evidence suggests that, on the same day, different subregions within a callus can be at different stages of repair and may be progressing at different rates. Given that, a parsimonious strategy is to select separated target regions within the same callus and develop simulations for each in sequence. They would be treated as independent modules. Future work utilizing separate simulations of independent target regions will help bring regional issues into focus prior to engineering their merger. The process of merging initially independent modules incrementally into a tissue healing whole would occur further downstream. Given that this work strives to establish the feasibility of the Fig 1 approach, it is more efficient to focus first on one target region.

References

1. Craver, Carl and Tabery, James, "Mechanisms in Science", The Stanford Encyclopedia of Philosophy (Fall 2016 Edition), Edward N. Zalta (ed.), forthcoming. http://plato.stanford.edu/archives/fall2016/entries/science-mechanisms/

2. Braillard PA, Malaterre C. Explanation in biology. An enquiry into the diversity of explanatory patterns in the life sciences 2015. Springer, Netherlands. http://www.springer.com/us/book/9789401798211

3. Bechtel W, Abrahamsen A. Explanation: A mechanist alternative. Studies in History and Philosophy of Science Part C: Studies in History and Philosophy of Biological and Biomedical Sciences. 2005;36: 421-41. http://www.sciencedirect.com/science/article/pii/S1369848605000269

4. Craver, C. F. (2007). Explaining the brain: Mechanisms and the mosaic unity of neuroscience, p.6. Oxford: Oxford University Press. https://global.oup.com/academic/product/explaining-the-brain-9780199299317

5. Illari PM, Williamson J. What is a mechanism? Thinking about mechanisms across the sciences. European Journal for Philosophy of Science. 2012;2: 119-35. http://link.springer.com/article/10.1007/s13194-011-0038-2

6. Braillard PA, Malaterre C. Explanation in biology: an introduction. In Braillard PA, Malaterre C. Explanation in biology. an enquiry into the diversity of explanatory patterns in the life science. 2015 (pp. 13-14). Springer Netherlands. http://link.springer.com/chapter/10.1007/978-94-017-9822-8_1

7. Craver CF. When mechanistic models explain. Synthese. 2006;153: 355-76. http://link.springer.com/article/10.1007/s11229-006-9097-x

8. Kaplan DM, Craver CF. The explanatory force of dynamical and mathematical models in neuroscience: A mechanistic perspective. Philosophy of Science. 2011;78: 601-27. http://www.jstor.org/stable/10.1086/661755?seq=1#page_scan_tab_contents

9. Darden L. Thinking again about biological mechanisms. Philos Sci. 2008;75: 958-69. http://www.journals.uchicago.edu/doi/abs/10.1086/594538

10. Darden L. Strategies for Discovering Mechanisms: Schema Instantiation, Modular Subassembly, Forward/Backward Chaining. Philos Sci. 2002;69(S3): S354-365. http://www.jstor.org/stable/10.1086/341858
